# Acute diquat poisoning in children: a single-center retrospective observational study

**DOI:** 10.3389/fped.2026.1844802

**Published:** 2026-07-30

**Authors:** Mingxia Li, Peisheng Jia, Erhu Wei

**Affiliations:** Department of Pediatric Intensive Care Unit, The First Affiliated Hospital of Zhengzhou University, Zhengzhou, China

**Keywords:** children, diquat poisoning, dose-response relationship, multiple organ dysfunction, prognosis

## Abstract

**Background:**

Acute diquat poisoning can cause multi-organ dysfunction and even death. The dose-effect relationship and multi-organ injury patterns of diquat poisoning in children remain unclear.

**Objective:**

This study analyzed 58 pediatric cases to systematically characterize clinical features and investigate the relationship between intake dose, organ injury patterns and prognosis.

**Methods:**

Patients were divided into three groups according to the volume of diquat ingested: group A (≤20 mL, *n* = 24), group B (21–50 mL, *n* = 15), and group C (>50 mL, *n* = 19). General clinical information, incidence of organ injury, laboratory tests at three time points (0–24 h [T1], 48–72 h [T2], and 5–7d [T3] after admission), and prognosis were compared.

**Results:**

Dose-dependent multi-organ injury was observed. Specifically, the incidences of acute kidney, liver, gastrointestinal and central nervous system injuries all increased significantly with the increase of dose (all *p* < 0.05). At the laboratory level, early inflammatory parameters and metabolic acidosis parameters also showed dose-dependent significant increases (all trends *p* < 0.05), and the indicators related to liver and kidney function injury reached their peak 48–72 h after admission. The incidence and severity of multiple organ damage in children with acute diquat poisoning are dose-dependent. Early severe inflammation and metabolic acidosis are key features of this condition. All seven fatalities occurred in group C, and the intake dose was 100–200 mL.

**Conclusions:**

Acute diquat poisoning in children appears to be dangerous, and the occurrence and severity of multiple organ damage are correlated with the intake dose. Early intense inflammation and metabolic acidosis appear to be important features of this disease.

## 1 Introduction

Diquat is a fast-acting herbicide structurally similar to paraquat. Due to its widespread application in agriculture, the incidence of acute diquat poisoning has been on the rise in recent years. Although classified as moderately toxic by the World Health Organization, acute diquat poisoning can lead to severe manifestations like acute central nervous system injury (1), acute kidney injury (2, 3), rhabdomyolysis (4, 5), multi-organ failure (6, 7), and even death (8, 9). The severity of poisoning is generally related to the dose consumed (10). Although diquat poisoning may produce similar manifestations in children and adults, treatment decisions may be affected by physiological and metabolic differences (5, 11). In pediatric populations, ingestion of diquat may cause liver or kidney damage (12), rhabdomyolysis and shock (13), and fetal outcomes (14), yet large-scale epidemiological evidence is scarce at home and abroad (5, 14–16). The dose-effect relationship, time course of organ damage, and key factors affecting prognosis remain unclear, which presents significant challenges in pediatric clinical decision-making. Based on the above background, we conducted a retrospective observational cohort study on 58 cases of acute diquat poisoning in children to address these gaps. We systematically summarized the clinical manifestations, laboratory and imaging characteristics of diquat poisoning in children, and focused on the relationship between the intake dose and multiple organ damage patterns, severity, and prognosis to provide an evidence-based basis for improving the diagnosis and treatment of diquat poisoning in children.

## 2 Materials and methods

### 2.1 Subjects

Clinical data of children with acute diquat poisoning admitted to the intensive care unit of our hospital between August 2020 and December 2024 were collected. The inclusion criteria were as follows: 1) age ≥28 days and <18 years; 2) visit to our hospital within 72 h after taking diquat; and 3) patients should be clinically diagnosed with diquat poisoning, and blood/urine toxicology analysis results do not detect mixed other drugs and poisons. The exclusion criteria were as follows: 1) patients with previous liver or kidney dysfunction, heart disease, diabetes, respiratory system disease, genetic metabolic disease, nervous system disease, and other underlying diseases; 2) patients who have been confirmed by toxicological analysis to have mixed exposure to other toxins/drugs; 3) patients with incomplete medical records.

### 2.2 Data collection

Clinical data of patients were collected, including general information, intake routes and doses, clinical manifestations and laboratory examination indicators, imaging findings, treatments (including use of hemoperfusion and hemodialysis), length of stay, treatment costs, and condition at discharge. Collect the laboratory parameters of the patients at three time points after admission: T1 (within 24 h after admission), T2 (48–72 h after admission), and T3 (5–7 days after admission). Evaluation method of poison dosage: The hospital routinely assesses ingestion amounts in oral liquid poisoning cases through two approaches: accurate dose measurement and simulated ingestion evaluation. Accurate dose measurement: The patient orally stated the milliliters of diquat solution taken orally, or based on the capacity of the poison bottle and the volume of the remaining poison. Simulated ingestion evaluation: The patient simulated the poisoning event without swallowing, and spat water into a measuring cylinder. This was repeated three times to obtain an average volume.

### 2.3 Definition of organ injury

Digestive tract injuries: oral mucosal ulcers, ruptures and bleeding, hemorrhagic fluid from gastrointestinal decompression, gastric retention, toxic intestinal paralysis, elevated intra-abdominal pressure, bloody stools, etc., (17). Renal injury: Elevated serum creatinine (sCr) ≥ 0.3 mg/dL (≥26.5 umol/L), or sCr reaching 1.5 times the baseline value; Or urine output is less than 0.5 mL·kg-1·h-1 for more than 6 h (18); Liver injury: Alanine aminotransferase (ALT) ≥ twice the normal reference value (19); Central nervous system injury: Patients may experience symptoms such as dizziness, fatigue, irritability, delirium, apathy, drowsiness, convulsions and coma, or imaging examinations may suggest the presence of brain parenchymal damage (20). Lung injury: Patients may experience chest tightness, shortness of breath, hypoxemia or respiratory failure, or chest CT may indicate exudative changes in the lungs, interstitial changes in the lungs, pleural effusion, etc (21). Cardiovascular injury: Symptoms such as heart failure or expansive shock, circulatory failure occur, or there are other abnormal changes such as elevated myocardial enzymes or decreased left ventricular ejection fraction indicated by echocardiography (21); Pancreatic injury: Serum amylase (Amy) and/or lipase levels increase by more than three times the upper limit of normal values; Or CT indicates peripancreatic exudation (22); Rhabdomyolysis: Creatine kinase (CK) > 1,000 U/L or CK > 5 times the upper limit of the normal reference value; Patients may be accompanied by symptoms such as muscle weakness, muscle pain and muscle swelling (23).

### 2.4 Statistical methods

We divided the patients into three groups based on the dosage of diquat intake for statistical analysis. These thresholds were selected based on previous literature (21, 24, 25), clinical feasibility and preliminary data review for inter-group comparison. GraphPad Prism 9.0 software was used for data analysis. Data were analyzed using the Shapiro–Wilk test for normality. Normal distribution data are expressed as mean ± SD, and group comparisons were performed using one-way ANOVA. Non-normally distributed data are expressed as median (Q1, Q3), and group comparisons were performed using the Kruskal–Wallis test. Count data are expressed as number (percentage) [*n* (%)], and Fisher's exact was used for comparison between groups. Univariate logistic regression analysis was used to explore the dose-prognosis relationship. Given the limited number of death events, the Firth's penalized likelihood was adopted for correction. *P* < 0.05 was considered statistically significant.

### 2.5 Ethical approval

This study was approved by the Ethics Committee for Scientific Research and Clinical Trials of the First Affiliated Hospital of Zhengzhou University (Approval No. 2024-KY-1431-001).

## 3 Results

### 3.1 Patient general information

58 children who orally ingested 20% diquat solution were included in the study. Among them, 25 were male (43.10%) and 33 were female (56.90%), aged 11–17 years (mean 14.55 ± 1.81 years). There were 12 patients (20.70%) with depression, anxiety or bipolar disorder, and 23 patients (39.66%) who took diquat due to temporary psychological factors such as quarrels with others, study pressure and emotional fluctuations. The intake dose of diquat ranged from 1 to 200 mL, with a median of 30.00 (10.00, 92.50) mL. The time from poisoning to visiting our hospital was 2–70 h, with an average of 8.00 (5.75, 16.00) hours. The average hospital stay was 7.50 (4.00, 11.25) days, and 51 patients (87.93%) survived, and 7 cases (12.07%) died. The hospital stay of the deceased patients was 4 h−5 d, with an average of 2 (2) days.

### 3.2 Clinical features

#### 3.2.1 Group characteristics and treatments

Patients were divided into three groups: ≤20 mL (group A), 21–50 mL (group B), and >50 mL (group C). There were no significant differences among the three groups in terms of age, sex, time to admission, or basic treatment regimens (gastric lavage, adsorption, catharsis, etc., all *p* > 0.05). However, the proportion of patients receiving blood purification treatment (hemoperfusion and/or dialysis) increased significantly with the increase in dose, with *p* values of 0.0097 and 0.0188, respectively. All 7 deaths occurred in group C, with a *P*-value difference of 0.0002 between the groups. The intake dose range of diquat for the deceased patients was 100–200 mL. Univariate logistic regression showed that the ingested dose was significantly associated with mortality (Firth -corrected OR = 1.0384, 95% CI: 1.0189–1.0744, *p* < 0.001). Hospital stay and cost showed significant overall differences among the three groups (*p* < 0.05), but no significant pairwise differences were found. Detailed grouping baseline characteristics and treatments are presented in Table 1.

**Table 1:** Baseline characteristics and treatment regimens of patients in three groups.

Indicator	Group A (*n* = 24)	Group B (*n* = 15)	Group C (*n* = 19)	*p*
Age (years)	13.96 ± 1.9	15 ± 1.51	14.95 ± 1.78	0.1088
Male	9 (37.5%)	8 (53.3%)	8 (42.1%)	0.6203
Time to admission (h)	12 (6.25,22.25)	10 (7,12)	6 (5,9)	0.0734
Gastric lavage	22	13	19	0.2832
Adsorption	13	7	12	0.6707
Catharsis	18	10	16	0.5068
Diuresis	22	13	19	0.2832
Antioxidant	23	15	19	>0.9999
Glucocorticoid	17	11	14	>0.9999
Hemoperfusion	17	14	19^a^	0.0097
Hemodialysis	5	7	12^a^	0.0188
Deaths	0	0	7^a^^,^^b^	0.0002
Hospital stays (d)	5.5 (4.0, 10.0)	11.0 (7.0, 15.0)	6.0 (2.0, 12.0)	0.0481
Hospitalization cost (¥)	18,580 (13,277, 36,069)	36,587 (22,874, 55,763)	35,009 (17,974, 55,861)	0.0411

Data are expressed as mean ± SD, median (IQR), or *n* (%).

a *P* < 0.05 versus Group A.

b *P* < 0.05 versus Group B.

#### 3.2.2 Incidence of organ injury

With the increase of the intake dose, the incidence of organ injury showed a dose-dependent increase. Among them, the differences in the incidence of injury in the digestive tract, kidneys, liver and central nervous system between groups were statistically significant. Especially, the intergroup differences in acute central nervous system injury were the most significant (*p* = 0.0007). Table 2 shows the organ damage in patients with different poison intake doses.

**Table 2:** Incidence of organ injury [*n* (%)] in children exposed to different doses of diquat.

Affected organs or systems	Group A	Group B	Group C	*p*
Digestive tract	17 (70.83)	14 (93.33)	19 (100.00)^a^	0.0097
Kidney	6 (25.00)	9 (60.00)a	14 (73.68)^a^	0.0043
Liver	1 (4.17)	6 (40.00)a	8 (42.11)^a^	0.0033
Central nervous system	3 (12.50)	5 (33.33)	13 (68.42)^a^	0.0007
Lung	9 (37.50)	8 (53.33)	13 (68.42)	0.1331
Cardiovascular system	3 (12.50)	3 (20.00)	6 (31.58)	0.3311
Pancreatic	2 (8.33)	3 (20.00)	6 (31.58)	0.1519
Rhabdomyolysis	0 (0.00)	1 (6.67)	3(15.79)	0.1178

a *P* < 0.05 versus Group A.

### 3.3 Dynamic changes of laboratory indicators

#### 3.3.1 Comparison of laboratory indicators within 24 h of admission (T1)

Inflammation-related indicators demonstrated a significant dose-dependent increase in white blood cell count (WBC). Group C [16.57 (13.71, 23.26) × 10⁹/L] exhibited a significantly higher WBC than group A [10.83 (8.48, 13.97)  ×  10⁹/L]. Additionally, group C's count was numerically greater than that of group B [12.77 (11.15, 17.53) × 10⁹/L]. The percentage of neutrophils (NEU%) also rose with increasing dose, with group C [92.0 (90.8, 94.0) %] significantly surpassing group A [85.7 (77.15, 90.45) %]. Conversely, the percentage of lymphocytes (Lymph%) exhibited an inverse trend; group C [4.7 (3.4, 5.8) %] was significantly lower than group A [8.15 (6.52, 19.2) %]. This finding indicates that high-dose diquat intake can elicit a robust inflammatory stress response in the early stages, closely correlating with the intake dose. Procalcitonin (PCT) revealed that both group B [0.093 (0.038, 0.200) ng/ml] and group C [0.075 (0.035, 0.336) ng/ml] were significantly elevated compared to group A [0.032 (0.021, 0.065) ng/ml]. This suggests that diquat poisoning can induce a systemic inflammatory response even in the absence of clear infection evidence, with effects manifesting early in the medium and high-dose groups. Furthermore, the level of interleukin-6 (IL−6) increased with dose; group C [14.49 (2.21, 68.06) pg/ml] exhibited a significantly higher level than group A [3.03 (1.5, 6.47) pg/ml], further confirming the dose-dependent mechanism of inflammatory factor release (Table 3).

**Table 3:** Comparison of laboratory parameters within 24 h of admission (T1).

Laboratory parameters	Group A	Group B	Group C	h	*p*
WBC ( × 10^9^/L)	10.83 (8.48, 13.97)	12.77 (11.15, 17.53)	16.57 (13.71, 23.26)^a^	17.30	0.0002
NEU% (%)	85.7 (77.15, 90.45)	89.6 (80.9, 94.2)	92 (90.8, 94)^a^	12.69	0.0018
Lymph% (%)	8.15 (6.52, 19.2)	4.6 (3.9, 12.7)	4.7 (3.4, 5.8)^a^	15.56a	0.0004
PCT (ng/mL)	0.032 (0.021, 0.065)	0.093 (0.038, 0.200)^a^	0.075 (0.035, 0.336)^a^	10.69	0.0048
IL-6 (pg/mL)	3.03 (1.5, 6.47)	5.27 (2.96, 37.11)	14.49 (2.21, 68.06)^a^	6.505	0.0387
CK(U/L)	75.5 (52.25, 142)	90 (64, 132)	147.5 (70.75, 217.5)	4.384	0.1117
ALT (U/L)	9.5 (9, 11)	16 (11, 27)^a^	13 (8, 58)	9.288	0.0096
AST (U/L)	18 (15, 22.25)	19 (16, 23)	20 (17, 36)	4.421	0.1097
Amy (U/L)	58 (38.5, 97)	73 (46.75, 105.5)	116 (105, 178)^a^^,^^b^	11.33	0.0035
BUN (mmol/L)	4.15 (3.24, 6.87)	5.2 (4, 7.8)	5.39 (3.6, 7.4)	4.060	0.1313
sCr (umol/L)	52.5 (40.5, 79.5)	78 (53, 145)	66 (51, 127)	5.109	0.0777
TP (g/L)	72.85 (68.85, 77.4)	78.1 (72.7, 83)	77.7 (71.8, 84.3)	5.139	0.0766
Alb (g/L)	46.75 (45.1, 48.78)	48.8 (47.2, 52.7)	49 (46, 52.2)	3.738	0.1543
Lac (mmol/L)	1.6 (0.85, 2.4)	1.95 (0.9, 2.72)	2.2 (1.65, 5.6)	3.338	0.1884
SBE (mmol/L)	−3.1 (−6.9, −1.9)	−6.1 (−8.4, −5.4)	−8.9 (−13.4, −5.55)^a^	9.793	0.0075

a *P* < 0.05 versus Group A.

b *P* < 0.05 versus Group B.

Among the liver function-related indicators, the comparison of ALT levels across the three groups revealed statistically significant differences (H = 9.288, *P* = 0.0096), with group B exhibiting levels significantly higher than those in group A. Additionally, the level of Amy demonstrated a significant dose-dependent increase. Group C [116 (105,178) U/L] was not only significantly higher than group A [58 (38.5,97) U/L], but also exceeded group B [73 (46.75,105.5) U/L] significantly, indicating that high-dose poisoning may affect pancreatic function.

Regarding acid-base balance indicators, the negative value of standard base excess (SBE) increased with the dosage. Group C [−8.9 (−13.4, −5.55) mmol/L] exhibited a significantly lower SBE than Group A [−3.1 (−6.9, −1.9) mmol/L], indicating severe metabolic acidosis that is directly correlated with the intake dose. This measure can serve as a crucial indicator for the early assessment of poisoning severity.

Other organ function-related indicators, including CK, aspartate aminotransferase (AST), blood urea nitrogen (BUN), and sCr, exhibited an increasing trend with dose at time point T1, though the inter-group differences were not statistically significant. This finding may be attributed to the early stage of poisoning, where organ damage had not yet fully manifested, consistent with the subsequent peak of organ injury observed at T2 (48–72 h post-admission).

#### 3.3.2 Comparison of laboratory indicators 48–72 h after admission (T2)

As the disease progressed, both WBC and NEU% declined compared to T1, with no statistically significant differences observed between the groups. This decline may be attributed to the body's self-regulation of the inflammatory response or to clinical intervention measures. Nevertheless, significant differences in lymphocyte percentages and PCT levels between the groups remained evident (H = 6.556, 7.206; *P* = 0.0377, 0.0272). Furthermore, PCT levels increased at T2 relative to T1, which might be related to secondary infection and multiple organ dysfunction (Table 4).

**Table 4:** Comparison of laboratory indicators at 48−72 h (T2) after admission.

Laboratory Parameters	Group A	Group B	Group C	h	*p*
WBC ( × 10^9^/L)	9.58 (8.36, 11.10)	11.72 (9.2, 14.99)	10.92 (7.51, 16.07)	2.485	0.2887
NEU% (%)	68.6 (63.15, 83.85)	79.7 (65.3, 89.4)	81.6 (75.43, 87.9)	4.475	0.1067
LYMPH% (%)	22.65 (12, 28.4)	12.3 (6.8, 26.2)	11.3 (7.32, 17.63)	6.556	0.0377
PCT (ng/mL)	0.041 (0.027, 0.15)	0.095 (0.029, 0.26)	0.32 (0.1, 42.23)^a^	7.206	0.0272
CK (U/L)	36 (24, 57)	106 (67.5, 235)^a^	42 (31, 113.5)	10.47	0.0053
ALT (U/L)	9.5 (5.5, 15.75)	14 (10, 42)	36 (10, 191)^a^	9.762	0.0076
AST (U/L)	16 (11, 23)	20 (14, 32)	36 (15, 90)^a^	6.701	0.0351
Amy (U/L)	36 (26, 77)	43 (24.75, 119)	64 (57.75, 185.8)	4.866	0.0833
BUN (mmol/L)	3.51 (2.98, 5.56)	7.8 (3.5, 11)^a^	9.31 (5.31, 11.43)^a^	14.72	0.0006
sCr (umol/L)	51 (39, 67.75)	89 (57, 266)^a^	119 (67.5, 225)^a^	18.31	0.0001
TP (g/L)	64.1 (60.95, 72.6)	62.9 (53.7, 64.5)	60.8 (52.7, 66.8)	3.373	0.1852
Alb (g/L)	39.3 (37.95, 42)	37.3 (31.7, 41.1)	35.3 (31.1, 42)	4.105	0.1284
Lac (mmol/L)	0.8 (0.5, 1.7)	1 (0.82, 3.32)	1.7 (1.45, 4.5)^a^	6.990	0.0304
SBE (mmol/L)	−0.65 (−5.12, 1.75)	−1 (−5.25, 2.77)	−4.1 (−8.4, −1.75)	3.413	0.1815

a *P* < 0.05 versus Group A.

The levels of liver function indicators ALT and AST in group C were significantly elevated compared to those in group A, indicating a time-cumulative effect of liver cell injury, with more severe damage observed in the high-dose group. Additionally, the levels of renal function indicators BUN and sCr in both group B and group C were significantly higher than those in group A, with group C exhibiting greater values than group B. This finding suggests that renal injury induced by diquat progressively developed during the disease course and demonstrated a dose-dependent relationship. Although the Amy level displayed an increasing trend with dosage, the differences among the groups were not statistically significant (*P* = 0.0833).

The indicators that initially exhibited no significant differences gradually demonstrated dose-related changes. The CK level in group B [106 (67.5, 235) U/L] was significantly elevated compared to group A [36 (24, 57) U/L], indicating the progression of skeletal muscle injury. Although the CK level in group C was greater than that in group A, the difference did not reach statistical significance.

The Lac level in group C [1.7 (1.45, 4.5) mmol/L] was significantly elevated compared to that in group A [0.8 (0.5, 1.7) mmol/L]. This finding suggests that patients experiencing high-dose poisoning may exhibit metabolic disorders, potentially linked to organ function impairment.

#### 3.3.3 Comparison of laboratory indicators 5–7 days after admission (T3)

In the later stages of the disease, the dose correlation of certain indicators remained evident. The levels of CK, sCr, and BUN all decreased compared to those observed at T2. The CK level in group B [83 (47.75, 216.3) U/L] remained significantly higher than that in group A [33 (28.25, 45.25) U/L]. Overall, the sCr level continued to increase with the dose (*P* = 0.0494), while the difference in BUN between the groups was no longer present (Table 5).

**Table 5:** Comparison of laboratory indicators on days 5–7 (T3) after admission.

Laboratory parameters	Group A	Group B	Group C	h	*p*
WBC ( × 10^9^/L)	8.29 (7.2, 10.89)	9.6 (9.04, 11.76)	8.8 (5.84, 12)	1.729	0.4214
NEU% (%)	66.4 (57.9, 81.7)	71.1 (60.1, 84.7)	69.7 (62.2, 76.2)	0.6171	0.7345
LYMPH% (%)	20.4 (11.7, 32.5)	22.8 (6.6, 27.5)	20.6 (13.5, 26.4)	0.8312	0.6600
PCT (ng/mL)	0.041 (0.037, 0.16)	0.13 (0.05, 0.33)	0.12 (0.03, 0.31)	2.121	0.3637
CK (U/L)	33 (28.25, 45.25)	83 (47.75, 216.3)^a^	51 (31.25, 196.8)	7.148	0.0280
ALT (U/L)	15 (13, 51.5)	39 (23, 56)	23 (18.75, 60.75)	1.447	0.4852
AST (U/L)	17 (14.75, 31.75)	30 (18, 44)	23.5 (15.75, 25.5)	4.633	0.0986
BUN (mmol/L)	4.59 (3.75, 6.17)	5.7 (4.2, 15.28)	6.25 (3.46, 7.5)	2.667	0.2636
sCr (umol/L)	52 (43, 75.08)	63 (50, 288)	77 (54.5, 146.3)	6.017	0.0494
TP (g/L)	62.3 (59.95, 66.95)	56 (49.2, 59.4)^a^	59.1 (55.83, 64.58)	9.306	0.0095
Alb (g/L)	37.55 (36.15, 40.53)	34.1 (31.5, 34.9)^a^	35.95 (33.18, 39.2)	12.23	0.0022
Lac (mmol/L)	1.1 (0.55, 1.8)	1.1 (0.75, 1.6)	1.15 (0.77, 1.77)	0.2982	0.8719
SBE (mmol/L)	0 (−2.45, 2.3)	−3.15 (−4.5, 1.57)	−1.3 (−3.6, 4.02)	2.019	0.3644

a *P* < 0.05 versus Group A.

The levels of total protein (TP) and albumin (Alb) exhibited a dose-dependent decrease. Specifically, the TP levels in group B [56 (49.2, 59.4) g/L] and Alb levels [34.1 (31.5, 34.9) g/L] were significantly lower than those in group A, indicating a decline in liver synthetic function and an increase in body consumption due to severe poisoning.

In contrast, other indicators, including WBC, NEU%, Lymph%, and PCT, did not demonstrate statistically significant differences among the groups at the T3 time point. This finding suggests that as the disease progresses, certain inflammatory indicators may stabilize, potentially reflecting the body's compensatory mechanisms and the effects of clinical treatment.

### 3.4 Imaging findings

**Figure 1:**
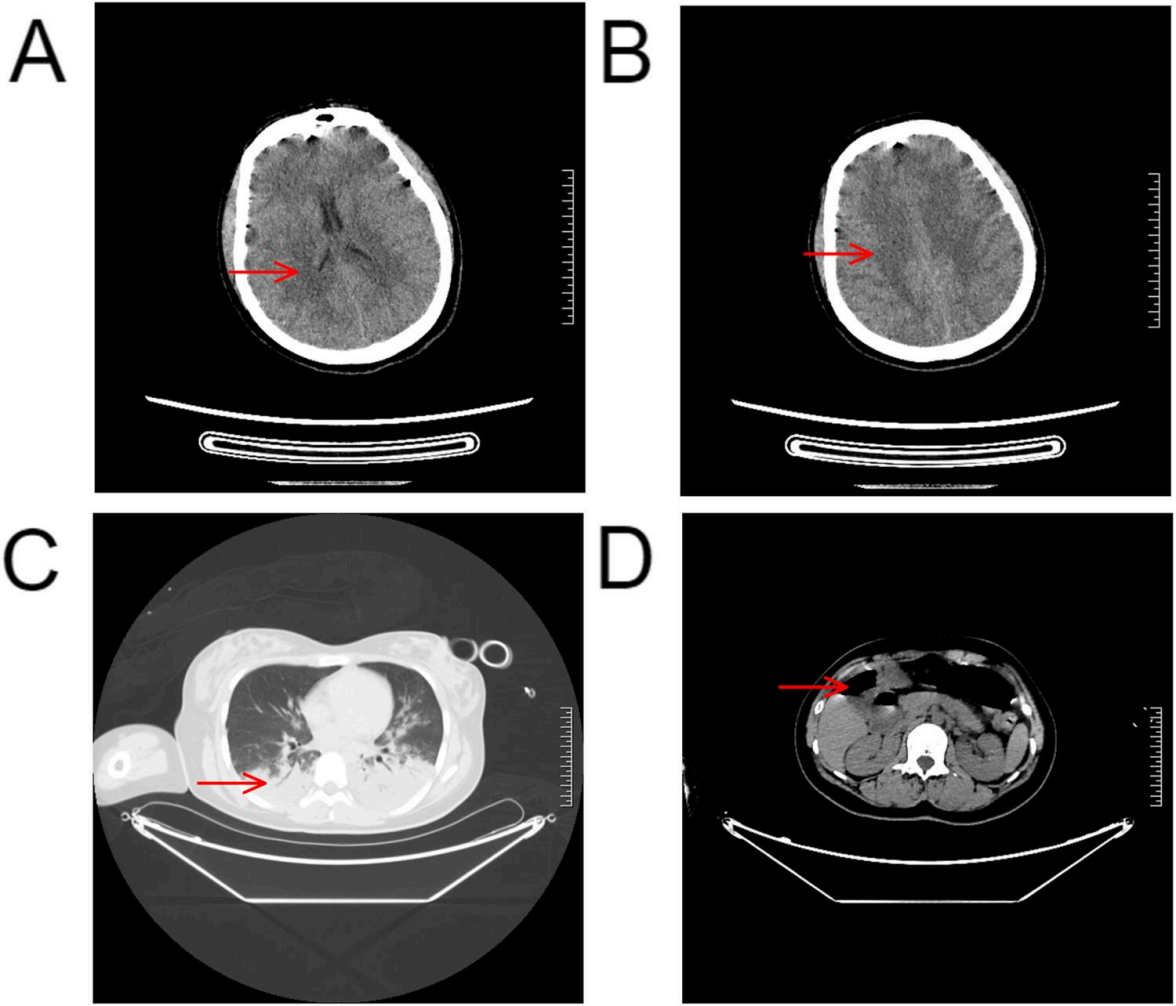
Plain CT scan of the head, chest, and abdomen of a 17-year-old girl on the day of admission. Both **(A)** and **(B)** exhibit blurred sulci and fissures in the bilateral cerebral hemispheres, diffuse edema of the brain tissue. **(A)** Red arrow indicates a symmetrical reduction in white matter density adjacent to the lateral ventricle. **(B)** Red arrow indicates a decrease in white matter density within the semi-oval center of the brain. **(C)** Red arrow points to exudation and consolidation in lung. **(D)** Red arrow shows intestinal dilation with accumulation of gas and fluid.

Imaging findings confirmed multi-organ involvement, most imaging studies were performed within 72 h after admission. Chest CT/x-ray (51patients): 21 were normal, 20 had inflammatory exudation-like changes, 12 had pleural effusion, and 8 had small nodules. No diffuse pulmonary fibrosis was observed. Echocardiography (40 patients): 35 were normal, 3 had pulmonary hypertension, and 3 had mild valvular regurgitation. No overt systolic cardiac dysfunction was noted. Abdominal imaging: Ultrasound (38 patients) showed renal echo enhancement in 7, hepatic echo enhancement in 4, and coarse gallbladder wall in 5. CT (15 patients) revealed intestinal dilatation/effusion in 4, uneven renal density in 2, and pelvic effusion in 4. Head CT/MRI (12 patients): 8 were normal. The primary abnormalities included cerebral edema and low-density lesions in the brainstem, thalamus, cerebellar hemispheres, and periventricular white matter. MRI revealed pontine and bilateral pontine lesions (patchy, slightly longer T2 and elevated DWI signals). Figure 1 shows the CT scan images of the head, chest and abdomen of a 17-year-old girl on the day of her admission. She came to our hospital 20 h after taking a whole bottle of 200 mL of diquat solution orally. As shown in Figure 1, (A) and (B) highlight the symmetrical reduction in white matter density adjacent to the lateral ventricles on both sides and within the bilateral semi-oval centers of the brain, (C) highlights pulmonary inflammatory exudative and consolidation, and (D) shows intestinal dilation with fluid and gas accumulation.

## 4 Discussion

This study addresses the limited evidence on acute diquat poisoning in children, which currently relies predominantly on case reports (5, 15). The mortality rate we observed was 12.07%, close to a previous report from our institution (15.29%) (21) but lower than historical rates of 43%–60% (24, 26). This discrepancy may be attributed to our strict exclusion of co-exposure to paraquat (commercial diquat often contains paraquat), a key methodological difference given the recognized high toxicity of paraquat (21, 27). The higher doses of diquat in adult studies than in pediatric studies may also be an important reason, with a mortality rate of 11.11% in Wu et al.'s. study at doses <50 mL (24). Different mortality rates may also be related to factors such as the time span studied, different geographical regions, and faster metabolism of children.

A dose-response relationship was observed in this study. Our study supports the claim that the toxic response of diquat is significantly dose-dependent, i.e., the higher the dose, the more severe the organ damage. This dose-dependent mortality pattern aligns with established toxicological principles. The rapid progression to death (mean 2 days) in these cases underlines the critical importance of early dose assessment for risk stratification.

Early markers of systemic disturbance were notable. Metabolic acidosis, and inflammatory markers were significantly elevated at T1 in a dose-dependent manner, preceding peak organ dysfunction. Metabolic acidosis may be associated with renal tubular damage caused by diquat through oxidative stress and pyroptosis (17, 28). Early elevated leukocytes, neutrophils, and IL-6 support the dual mechanisms of direct oxidative damage and secondary inflammation in diquat pathophysiology (17, 29, 30). These findings provide a physiological basis for early aggressive resuscitation and potential anti-inflammatory intervention (29, 31).

The temporal evolution of organ dysfunction followed a predictable pattern, with hepatic and renal injury peaking at 48–72 h after admission. This timeline corresponds with diquat's toxicokinetic profile (32) and suggests a defined window for therapeutic intervention. The absence of cardiac functional impairment despite circulatory failure may suggest central-mediated shock rather than primary myocardial depression, or it may be related to the timing of cardiac echocardiography. Neurological manifestations (brain edema, symmetrical thalamic and pontine lesions) consistent with previous reports (9, 33) reinforce central nervous system vulnerability. No pulmonary fibrosis was found, which supports that diquat has a lower risk of pulmonary fibrosis (34).

Although our inclusion criteria permitted children as young as 28 days, the final cohort comprised only adolescents aged 11 to 17. This discrepancy may be attributed to the infrequency of accidental ingestion of concentrated diquat among young children, coupled with the predominance of cases in this series involving intentional self-poisoning linked to psychological stress, a phenomenon that is rare in early childhood. Throughout the research period, no eligible young children were identified. Adolescents experience rapid physical and psychological changes, which can lead to emotional impulsiveness, diminished resilience to setbacks, and an inclination toward risky behaviors. Education departments, families, and society should address the issue of pesticide poisoning among adolescents. It is essential to focus on children's psychological and behavioral well-being, enhance communication with adolescents, understand their psychological trends, and provide timely guidance and support. Additionally, there should be an emphasis on internet safety education and daily supervision for young people to improve their ability to discern risks and enhance their self-protection awareness, thereby reducing their exposure to and purchase of toxic substances. E-commerce platforms should be urged to enforce real-name certification rigorously, strengthen the review process for pesticide products, prohibit sales to minors, and clearly label product hazards and usage precautions. Furthermore, relevant authorities must intensify efforts to combat illegal sales and enhance market supervision.

The study has several limitations. First, its single-center, retrospective design introduced potential selection bias. Second, while the sample size is relatively large for pediatric diquat poisoning, it remains insufficient to analyze rare complications (e.g., rhabdomyolysis) and subgroup comparisons. Third, early deaths in critically ill patients led to missing data on laboratory indicators and imaging findings, which may underestimate the severity of organ damage in the high-dose group. This phenomenon underscores the severity of the disease and presents an inherent challenge in clinical research pertaining to critical care. Fourth, the usage rate of blood purification therapy was significantly higher in the high-dose group, which reflects that in clinical practice, more severe poisoning cases receive more aggressive treatment. This might have confused the relationship between dosage and outcome. Due to the limited number of deaths, we were unable to make reliable multivariate adjustments. Larger-sample prospective studies will be needed in the future to separate therapeutic effects from dose-effects. Fifth, the lack of long-term follow-up data and quantitative monitoring of diquat levels affects accurate toxicokinetic modeling. Finally, our dose-group thresholds are artificially defined rather than optimal predictive cut-offs found by statistical methods, and need to be validated by statistical optimization of larger cohorts. These limitations necessitate cautious interpretation of the results, particularly negative findings.

This study suggests dose-dependent organ injury patterns and identifies potential early metabolic markers in pediatric acute diquat poisoning. We hypothesize that early anti-IL-6 therapy might help alleviate multi-organ injury caused by severe poisoning. Future research should prioritize quantification of diquat levels to help establish toxicokinetic-pharmacodynamic relationships.

## Data Availability

The original contributions presented in the study are included in the article/Supplementary Material, further inquiries can be directed to the corresponding author.
